# Discrepancy between desired time in bed and desired total sleep time in patients with cancer: The DBST index and its relationship with insomnia severity and sleep onset latency

**DOI:** 10.3389/fpsyt.2022.978001

**Published:** 2023-01-11

**Authors:** Eulah Cho, Jaeeun Song, Joohee Lee, Inn-Kyu Cho, Dongin Lee, Hayun Choi, Harin Kim, Seockhoon Chung

**Affiliations:** ^1^Department of Psychiatry, Asan Medical Center, University of Ulsan College of Medicine, Seoul, South Korea; ^2^University of Ulsan College of Medicine, Seoul, South Korea; ^3^Department of Psychiatry, Veteran Health Service Medical Center, Seoul, South Korea; ^4^Department of Psychiatry, Seoul National University College of Medicine, Seoul, South Korea

**Keywords:** cancer, insomnia, sleep, circadian, the DBST index

## Abstract

Patients with cancer can often experience insomnia or sleep disturbances. This study aimed to explore whether the discrepancy between a patient’s desired time in bed and desired total sleep time (DBST index) can be used as a measurement tool for insomnia severity or sleep onset latency [SOL] in patients with cancer. This retrospective medical records review study gathered clinical information and scores from scales and indices such as the Insomnia Severity Index (ISI), Cancer-related Dysfunctional Beliefs about Sleep (C-DBS) scale, Patient Health Questionnaire-9 items (PHQ-9), State subcategory of State and Trait Anxiety Inventory, and the short form of the Fear of Progression Questionnaire. Sleep indices of time variables (bedtime, sleep onset time, and wake-up time), duration variables [SOL, time in bed (TIB), time in bed over 24 hours (TIB/d), and duration from wake-up time to bedtime (WTB)], and DBST index were calculated. ISI scores were predicted by the PHQ-9 (β = 0.34, *P* < 0.001), C-DBS scale (β = 0.17, *P* = 0.034), and DBST indices (β = 0.22, *P* = 0.004). Long SOL value was predicted by early bedtimes (β = −0.18, *P* = 0.045), short WTB durations (β = -0.26, *P* = 0.004), and high DBST index values (β = 0.19, *P* = 0.013). The DBST index was significantly correlated with both insomnia severity and SOL in patients with cancer.

## Introduction

Insomnia could be defined using different perspectives; however, in the context of symptoms, almost one-third of individuals experience insomnia as defined by the criteria of the Diagnostic and Statistical Manual of Mental Disorders-IV (DSM-IV) (1). It has been shown in many studies that behavioral and cognitive factors are involved in insomnia as well as biological factors (2). Some individuals could be treated for insomnia within a few days to weeks, while in other patients, symptoms can progress to chronic insomnia, which can last for several years. If the majority of insomnia causes are based on biochemical factors, most pharmacological treatments ought to be successful in all patients. However, even the British Association for Psychopharmacology consensus statement advises cognitive behavior therapy for insomnia (CBT-I) as the first choice for the short-term treatment of chronic insomnia (3). Moreover, according to the guidelines of the USA based on a systematic review, CBT is supported by moderate evidence in different age groups (4). Several studies have shown that CBT-I is effective in reducing sleep disturbance (4, 5) and the number of dysfunctional beliefs associated with sleep (6). Dysfunctional beliefs regarding sleep contribute to sleep disturbances and aggravate sleep problems. These include beliefs regarding the immediate consequences, long-term consequences, and control of sleep (7). To measure this concept, the Dysfunctional Beliefs and Attitudes about Sleep Scale (DBAS) is commonly used in clinical fields in several different questionnaire versions.

Insomnia is an important psychiatric issue for patients with cancer that does not seem to be managed easily. A longitudinal study showed that 74% of patients experience insomnia for a period of at least a year, and 46% report insomnia over a period of three years (8). A cross-sectional study found that approximately 43% of patients with cancer complained of insomnia, and 32% of patients with insomnia reported severe insomnia (9). The risk factors of insomnia and its correlation with cancer-related symptoms have not yet been elucidated. However, cancer, its treatment process, and its related psychological statuses could affect one another, and this relationship could be the cause of the existing distorted cognition associated with cancer, adding to the patients’ fear of death. Notably, patients with cancer report fairly sensible dysfunctional beliefs regarding insomnia, with statements such as “If I don’t get enough sleep, my immune system will be broken down and then I will get sick” (10). In a medical records review study in Korea, dysfunctional beliefs about sleep in patients with cancer were correlated with the severity of insomnia, regardless of their depression status (11). However, physicians pay limited attention to these beliefs, preferring to instead examine other accompanying symptoms such as pain (12). Untreated insomnia affects the caregivers and family of patients as well as the patients themselves, and it could pose an additional burden to the treatment process. Several studies suggest that insomnia can have a deleterious impact on the physical symptoms, social wellbeing, survival rates, and relapse rates in patients with cancer (13). Therefore, managing insomnia and improving sleep is essential in patients with cancer, as is the administration of other major treatments such as chemotherapy or radiotherapy (14).

The discrepancy between the desired time in bed and desired total sleep time (DBST index) refers to the discrepancy between one’s desired total hours in bed and one’s desired hours of total sleep (15, 16). One of the most popular misbeliefs of patients with insomnia is that the longer the sleeping time, the greater the health benefits. People need only a certain amount of sleep every 24 h, and the average amount of sleep needed gradually decreases over the course of one’s life span (17). According to the two-process model, sleep is regulated by the interaction between process S (the homeostatic drive) and process C (the circadian process). Process S relates to the idea that the longer the period of one’s arousal, the more they need to sleep. This can be referred to as one’s ‘sleep pressure’, which is significantly associated with the circadian rhythm that is controlled by the suprachiasmatic nuclei (SCN). This model supports non-pharmacological treatment paradigms in psychiatry that are based on manipulating one’s circadian phase, sleep, and light exposure (18).

Discrepancies between actual times and reported/desired times pose an issue in studying insomnia. Several studies were previously interested in the concept of “paradoxical insomnia.” (19, 20). The polysomnographic (PSG) findings of paradoxical insomnia patients show significantly longer total sleep times than the patients’ reported sleep times (21). As an extension of this concept, a higher DBST index reflects that one wants to sleep for a duration greater than what they actually need. We hypothesized that the more severely individuals experience sleep disturbances, the larger this discrepancy would be.

We previously reported that the DBST index may be an index for insomnia severity (15, 16). Among the general population (15), the DBST index was significantly correlated with insomnia severity and preoccupation with sleep. In addition, a persistent preoccupation with sleep partially mediated the influence of the DBST index on the insomnia severity of patients. In another study conducted among the general population (16), the DBST index was significantly correlated with insomnia severity, depression, preoccupation with sleep, and dysfunctional beliefs about sleep. Additionally, a mediation analysis showed that the DBST index directly influenced the severity of insomnia, and depression, dysfunctional beliefs about sleep, and preoccupation with sleep mediated the association between the DBST index and severity of insomnia.

However, to date, there is no study on the DBST index among patients with cancer who experience insomnia or sleep disturbance due to the disease itself, its symptoms, the side effects of the existing treatment modalities, or fear of progression of the disease; thus, assessing the severity of insomnia is important for psychologically supporting patients with cancer. In this study, we aimed to explore whether the DBST index can be a measurement tool for determining the severity of insomnia in patients with cancer. The hypotheses of this study are that (1) the DBST index is associated with insomnia severity among patients with cancer, and (2) the DBST index is associated with sleep onset latency.

## Materials and methods

### Participants and procedure

This was a retrospective medical records review study of patients with cancer who visited the sleep clinic at the Asan Medical Center, which is specialized for patients with cancer, for the first time. We cross-sectionally reviewed the medical records of the first visits of patients with cancer who visited between May 1, 2021 and March 15, 2022. We selected a total of 146 patients after excluding those who met the following exclusion criteria: (1) patients who were immobile; (2) patients who had severe medical problems or brain metastases that influenced their cognitive function; (3) patients with severe psychotic symptoms or delirium; (4) patients who could not respond to the questionnaires; and (5) patients who had communication difficulties. At the first visit, a psychiatrist (a sleep specialist, S.C.), who was trained to manage cancer patients’ psychological problems, routinely evaluated patients’ psychiatric diagnoses through clinical interviews. Information regarding the age, sex, types of cancer, staging, current treatment modalities, responses to questions on sleep patterns, and rating scale scores of patients was collected. The protocol of this study was approved by the Asan Medical Center Institutional Review Board (2022-0353), and the requirement for informed consent was waived.

### Measures

#### Sleep indices and discrepancy between desired time in bed and desired total sleep time

When patients with cancer initially visited our sleep clinic, we routinely determine their sleep index scores by asking questions such as: “What is your usual bedtime?”; “What is your usual time to fall asleep?”; “What is your usual time to finally get out of bed in the morning?”; “For how many hours do you want to sleep in a day?”; and “From when until when do you want to sleep?” These data were routinely recorded in the patients’ medical records. On the basis of the patients’ responses to the questions pertaining to sleep patterns, we measured their sleep indices of time variables (bedtime, sleep onset time, and wake-up time), duration variables [sleep onset latency (SOL), time in bed (TIB), and duration from wake-up time to bedtime (WTB)] (22), and DBST index.

#### 1) Calculation of time variables

Time variables were obtained by averaging the usual times reported by patients. If a patient answered that they usually went to bed between 22:00 and 23:00, we estimated their usual bedtime to be 22:30 by averaging those times. For the statistical analysis, we transformed the usual times to numerical variables. A period of 15 min (one quarter of 1 h) was transformed into 0.25 (one quarter), while 30 min (half of 1 h) was transformed into 0.50. Therefore, 22:15 was transformed into 10.25.

#### 2) Calculation of duration variables

Duration variables were estimated using the time variables. SOL durations were estimated using the differences between participants’ sleep onset times and bedtimes, and their TIB durations were estimated on the basis of the differences between their wake-up times and bedtimes (TIB = wake-up time + 12 - bedtime). We also estimated the patients’ WTB, as it was reported that the WTB was significantly different between insomnia patients with sleep latency times ≤30 and >30 min (22). WTB durations were estimated using the duration from the time patients woke up to their bedtime (i.e., WTB = 24 – TIB) (22).

#### 3) Calculation of discrepancy between desired time in bed and desired total sleep time

The DBST index was estimated as the difference in the patient’s desired TIB and desired total sleep time (TST). Desired TST was estimated on the basis of responses to the question “For how many hours do you want to sleep in a day?” The desired TIB was estimated using responses to the question “From when until when do you want to sleep?” Finally, the DBST index was calculated as [desired hours of TIB] - [desired hours of TST] (15).

### Rating scales

#### 1) Insomnia Severity Index

The ISI is a self-rating scale that was developed to measure the severity of insomnia of an individual (23). A higher total score reflects a more severe level of insomnia. In this study, we applied the Korean version of the ISI scale (24).

#### 2) Cancer-related Dysfunctional Beliefs about Sleep scale

The Cancer-related Dysfunctional Beliefs about Sleep (C-DBS) is a self-reported rating scale that can measure cancer patients’ cancer-related dysfunctional beliefs related to sleep (10). It consists of two items that can be scored using a scale of 0–10. A higher total score reflects a higher level of dysfunctional beliefs about sleep. The C-DBS scale was originally developed in the Korean language, and we applied the original version in this study.

#### 3) Patient Health Questionnaire-9 items

The Patient Health Questionnaire-9 items (PHQ-9) is a self-rating scale that can measure the severity of depression in an individual (25). It consists of nine items that can be rated on a 4-point Likert scale that ranges from 0 (not at all) to 3 (nearly every day), with higher total PHQ-9 scores reflecting higher levels of depression. In this study, we applied the Korean version of the PHQ-9 (26).

#### 4) State subcategory of the State and Trait Anxiety Inventory

The State subcategory of the State and Trait Anxiety Inventory (STAI) is a self-rating scale that was developed for assessing patients’ traits and states of anxiety. It consists of 40 items, and these are categorized into two parts: 20 items for state assessments and the remaining 20 for trait assessments (27). The items can be rated on a 4-point Likert scale that ranges from 1 (not at all) to 4 (very much so). In this study, we applied the 20 items pertaining to participants’ states of anxiety from the Korean version of the scale (28).

#### 5) Short form of the Fear of Progression Questionnaire

The Short form of the Fear of Progression Questionnaire (FoP-Q-SF) is a shortened version of the original FoP-Q scale (29) that can measure the fear of disease progression in an individual, and it has been previously applied in cancer patients (30). The FoP-Q-SF consists of twelve items that can be rated on a 5-point Likert scale, with a higher total score reflecting a greater fear of progression. We applied the Korean version in this study (31).

#### 6) Assessment of pain using the numeric rating scale

The pain of cancer patients was assessed using a single-item numeric rating scale (NRS) (32).

### Statistical analysis

Data on the demographic characteristics and rating scale scores of patients were summarized as the means ± standard deviations. The level of significance for the analyses was defined as two-tailed at values of *P* < 0.05. In this study, we aimed to explore the usefulness of the DBST index in predicting the insomnia severity or sleep onset latency of patients. We divided the clinical variables into two parts: (1) rating scale scores and the DBST index and (2) sleep indices and the DBST index. In the first part, Pearson’s correlation coefficients were examined to explore the association of insomnia severity with other rating scales and the DBST index. A linear regression analysis employing stepwise methods and data-driven approaches was performed to explore whether the DBST index was associated with insomnia severity. In the second part, Pearson’s correlation analysis was performed to explore the associations among sleep onset latency and the other sleep indices, including the DBST index. A stepwise linear regression analysis was performed to explore whether the DBST index was associated with sleep onset latency. Statistical analysis was performed using SPSS version 21.0 (IBM Corp., Armonk, NY, USA) and Jamovi version 1.6.18.0.

## Results

A total of 146 patients were included in the analysis (Supplementary Table 1). Most patients (*N* = 109, 74.7%) were diagnosed with insomnia disorder. The others were diagnosed with depressive disorders (11.6%), anxiety disorders (5.5%), adjustment disorders (2.1%), and others. Regarding cancer-related variables, 137 patients (93.8%) had solid tumors and 17.2% had stage IV cancer (among patients with available TNM classification data). A total of 28.8% of patients had undergone surgery within three months. Regarding sleep indices, the means of the time and duration variables are shown in Supplementary Table 2. There were no significant differences in the DBST index values based on cancer types (breast cancer or gastrointestinal-hepatobiliary-pancreatic cancer *vs*. others, *P* = 0.393), cancer stages (stage IV *vs.* others among cancers with TNM staging data, *P* = 0.121), current classical chemotherapy (*P* = 0.083), receipt of a surgical procedure within three months of the study (*P* = 0.457), administration of radiation therapy (*P* = 0.479), or administration of immune or targeted therapy (*P* = 0.244). Among breast cancer patients, there was no significant difference in the DBST index between patients who were undergoing hormonal therapy and those that were not (*P* = 0.568). Among the different psychiatric diagnoses (insomnia disorder, depression *vs.* others), there were no significant differences in the DBST index (*P* = 0.969).

### Discrepancy between desired time in bed and desired total sleep time: Relations with psychological symptoms among cancer patients

Patient age was correlated with ISI (*r* = 0.26, *P* < 0.001) and FoP-Q-SF values (*r* = −0.16, *P* < 0.001) (Table 1). ISI scores were significantly correlated with the PHQ-9 (*r* = 0.37, *P* < 0.001), C-DBS (*r* = 0.24, *P* = 0.002), and DBST indices (*r* = 0.19, *P* = 0.020). DBST index scores were only significantly correlated with ISI scores (r = 0.19, *P* = 0.020, Supplementary Figure 1A).

**TABLE 1:** Correlation coefficients among rating scale scores and the DBST index among patients with cancer (*n* = 146).

Variables	Age	ISI	PHQ-9	STAI-S	FoP	C-DBS	Pain, NRS
ISI	0.26**						
PHQ-9	0.13	0.37**					
STAI-S	–0.03	0.12	0.45**				
FoP	−0.16**	0.10	0.64**	0.45**			
C-DBS	–0.03	0.24**	0.28**	0.16	0.41**		
Pain, NRS	0.03	0.14	0.27**	0.16	0.24**	0.19*	
DBST index	0.13	0.19*	–0.04	–0.03	–0.14	–0.08	0.03

ISI, Insomnia Severity Index; PHQ-9, Patient Health Questionnaire-9 items; STAI-S, state category of the State and Trait Anxiety Inventory; FoP, Fear of Progression questionnaire; C-DBS, Cancer-related Dysfunctional Beliefs about Sleep scale; DBST, discrepancy between desired time in bed and desired total sleep time.

*P < 0.05, **P < 0.01.

A stepwise linear regression analysis was conducted to explore which variables among age, PHQ-9, STAI-S, FoP-Q-SF, C-DBS, pain NRS, and DBST scores were associated with ISI scores (Table 2). PHQ-9 (β = 0.34, *P* < 0.001), C-DBS (β = 0.17, *P* = 0.034), and DBST index scores (β = 0.22, *P* = 0.004) were found to be associated with insomnia severity (adjusted *R*^2^ = 0.19, *F* = 12.5, *P* < 0.001).

**TABLE 2:** Stepwise linear regression analysis to explore the variables associated with insomnia severity in patients with cancer (*n* = 146).

Dependent variables	Included parameters	Beta	*P*-value	Adjusted R^2^	*F*, *P*-value
	PHQ-9	0.34	<0.001		
Insomnia Severity	C-DBS	0.17	0.034	0.19	*F* = 12.5
Index					*P* < 0.001
	DBST index	0.22	0.004		

The values were adjusted according to the age and scores from the state category of the State and Trait Anxiety Inventory and Fear of Progression scale. PHQ-9, Patient Health Questionnaire-9 items; C-DBS, Cancer-related Dysfunctional Beliefs about Sleep-2 items scale; DBST, discrepancy between desired time in bed and desired total sleep time.

### Discrepancy netween desired time in bed and desired total sleep time: Relations with sleep indices among cancer patients

Correlation analysis showed that a long SOL was significantly associated with a long TIB duration (*r* = 0.35, *P* < 0.001), short WTB (*r* = −0.35, *P* < 0.001), early bedtime (*r* = −0.32, *P* < 0.001), late sleep onset time (*r* = 0.54, *P* < 0.001), a high DBST index (*r* = 0.23, *P* = 0.005, Supplementary Figure 1B), and short desired TST (*r* = −0.17, *P* = 0.039) (Table 3). We conducted a stepwise linear regression analysis to explore the variables associated with long sleep onset latency durations. We included age, bedtime, WTB, and the DBST index as the variables in the final model as the wake-up time, TIB, desired TST, and desired TIB could influence the multicollinearity of the analysis. The stepwise regression model revealed that long SOL durations were associated with early bedtimes (β = −0.18, *P* = 0.045), short WTB values (β = −0.26, *P* = 0.004), and high DBST index scores (β = 0.19, *P* = 0.013) (adjusted *R*^2^ = 0.17, *F* = 10.5, *P* < 0.001, Table 4).

**TABLE 3:** Correlation coefficients of the DBST index and time/duration variables among patients with cancer (*n* = 146).

Variables	Age	SOL	TIB	WTB	Bedtime	Sleep onset time	Wake-up time	DBST index	Desired TIB
SOL	–0.06								
TIB	–0.16	0.35**							
WTB	0.16	−0.35**	−0.43**						
Bedtime	−0.18*	−0.32**	−0.25**	0.48**					
Sleep onset time	−0.20*	0.54**	–0.04	0.14	0.63**				
Wake-up time	−0.32**	0.10	0.24**	−0.66**	0.34**	0.38**			
DBST index	0.14	0.23**	–0.08	–0.06	–0.13	0.07	–0.05		
Desired TIB	–0.03	0.24	0.14	−0.28**	–0.15	–0.11	0.17*	0.29**	
Desired TST	–0.14	−0.17*	0.18*	−0.19*	–0.16	–0.16	0.19*	−0.59**	0.60**

SOL, sleep onset latency; TIB, time in bed; WTB, duration from wake-up time to bedtime; DBST, discrepancy between desired time in bed and desired total sleep time; TST, total sleep time.

*P < 0.05, **P < 0.01.

**TABLE 4:** Stepwise linear regression analysis to explore the predictive variables of sleep onset latency in patients with cancer (*n* = 146).

Dependent variables	Included parameters	Beta	*P*-value	Adjusted R^2^	*F*, *P*-value
	Bedtime	–0.18	0.045		
Sleep onset latency	WTB	–0.26	0.004	0.17	*F* = 10.5 *P* < 0.001
	DBST index	0.19	0.013		

The values were adjusted according to the values of age and time in bed over 24 hours. WTB, duration from wake-up time to bedtime; DBST, discrepancy between desired time in bed and desired total sleep time.

## Discussion

In this study, we attempted to explore whether the DBST index could be used as a measurement tool to determine insomnia severity in patients with cancer, and we hypothesized that the DBST index may be associated with (1) the severity of insomnia among patients with cancer, and (2) their SOL durations. We observed that the DBST index was significantly correlated with the predictive factors of both insomnia severity and SOL in patients with cancer.

### Insomnia severity and discrepancy between desired time in bed and desired total sleep time among cancer patients

As hypothesized, we observed that the DBST index was associated with the insomnia severity of patients with cancer in this study. Furthermore, the severity of insomnia in patients with cancer was also associated with the PHQ-9 and C-DBS scores. Patients with cancer often worry that their sleep disturbances may influence their cancer recurrence rate and impair their immune function (10), and they desperately want to sleep for at least 6.6 h (6.6 ± 1.2 h, Supplementary Table 2). This desire is influenced by these patients’ cancer-related dysfunctional beliefs about sleep. The C-DBS scale was developed to assess the dysfunctional beliefs about sleep among patients with cancer by questioning them with statements such as “My immune system will have serious problems if I don’t go to sleep at a certain time” (Q1-immune) and “If I don’t sleep well at night, my cancer may recur or metastasize” (Q2-recurrence) (10).

Patients with cancer may experience cancer-related fatigue symptoms due to chemotherapy and their physical or psychological states (33), and they unconsciously try to sleep for more time by laying on their bed or sofa. This might increase their desired TIB, and the discrepancy might increase their DBST index score. Paradoxically, this discrepancy reflects the severity of their insomnia, and spending a long time in bed during the daytime and having an early bedtime may aggravate the severity of their insomnia. Therefore, the CBT-I approach should be employed in these patients, as this approach was adapted for patients with cancer (34) and focuses on cancer patients’ cancer-specific precipitating factors based on 3P model of insomnia among cancer patients proposed by literatures (34, 35). These worrisome factors in patients can include daytime fatigue or pain, cancer-related dysfunctional beliefs about sleep, and fear of progression, all of which can influence patients’ sleep problems. Further study is needed to examine whether decreasing this discrepancy may reduce patients’ insomnia severity.

Parallel to the previous study, we observed that the C-DBS score was one of the factors associated with insomnia severity in this study. Additionally, we considered that the DBST index of patients with cancer might be influenced by their dysfunctional beliefs about sleep. However, we did not observe this association between the DBST index and C-DBS score. In our previous study (15), the DBST index was significantly correlated with insomnia severity and preoccupation with sleep (as measured using the Glasgow Sleep Effort Scale) but not with dysfunctional beliefs about sleep (as measured using the Dysfunctional Beliefs about Sleep-2 items scale) among the general population.

The lack of correlation between the DBST index and dysfunctional beliefs about sleep in the above studies can be explained. First, the DBST index might not accurately reflect participants’ dysfunctional beliefs about sleep. One of the core dysfunctional beliefs about sleep is that patients think that they should sleep more (e.g., “I must get 8 hours of sleep to feel refreshed and function well the next day”) (36). However, DBST index scores result from the discrepancies between patients’ desperate and “consciously reduced” desired sleep durations and their “unconscious” durations in bed. They do not calculate their desired time in bed on the basis of their desired total sleep time. Therefore, they decide their time in bed in accordance with their total sleep time; i.e., there should be no discrepancy. Therefore, this discrepancy might not reflect the classical dysfunctional beliefs about sleep. Second, the C-DBS scale, which was applied to measure cancer-related dysfunctional beliefs in this study, might not accurately reflect the dysfunctional beliefs of patients with cancer in a certain component linked with the DBST index. In another previous study (16), the DBST index was significantly correlated with the Dysfunctional Beliefs and Attitudes about Sleep-16 items scale scores, one of the most popularly applied rating scales (36).

The association between the DBST index and depression needs to be explored further, as we could not identify the correlation in the current study parallel to that in a previous study (15), despite significant findings in another study (16). The correlation might depend on the participants’ characteristics and should be elucidated in a further study investigating the DBST index among the clinical samples of patients with insomnia. Notably, pain is also an important factor for insomnia among cancer patients (37). However, in this study, the pain NRS was not significantly correlated with insomnia severity and the DBST index of patients; on the other hand, it was correlated with their depression, fear of progression, and cancer-related dysfunctional beliefs about sleep. The mean pain NRS score was relatively low (3.8 ± 2.9, range - 0 ∼ 10) in this study, and we consider the low level of pain to be one of reasons for the non-significant results that were contrary to our expectations.

### Sleep onset latency and discrepancy between desired time in bed and desired total sleep time among cancer patients

In this study, we explored the relationship between the DBST index and SOL. We hypothesized that the DBST index of patients with cancer may be associated with long SOL durations. We observed that a long SOL was associated with a high DBST index, late sleep onset time, and short WTB. This demonstrates that the DBST index can be used to assess the initiation of insomnia in cancer patients, as we observed an association between the DBST index and insomnia severity.

The concept of the DBST index was based on the idea that insomnia patients often go to bed early in the evening. They dysfunctionally believe that an early bedtime can induce an earlier sleep onset (38, 39). In addition, patients with cancer sometimes want to go to bed early in the evening, as they may experience cancer-related fatigue symptoms or the treatment modalities they are undergoing. However, according to the two-process model (40), an early bedtime does not guarantee an early sleep onset time. Sleep can be regulated by the interaction of a homeostatic drive and circadian rhythm, i.e., sleep can be forced by prolonged wakefulness (process S) and circadian timing pressures to fall asleep (process C).

The WTB index can be useful to explore the sleep-wake pattern of patients. In our previous study (22), a short sleep latency was correlated with a long WTB (16.5 h in the SOL ≤ 30 min group and 15.8 h in the SOL > 30 min group). In the current study, a long SOL was significantly correlated with a short WTB or early bedtime. This reflects that, on the basis of the two-process model of sleep regulation, a low homeostatic drive (presenting as a short WTB) and early circadian timing (presented as an early bedtime) cannot shorten a patient’s sleep latency. Compared to ISI scores, SOL durations can be considered as relatively objective indices, as these can be influenced by one’s circadian timing and homeostatic drive. This demonstrates that the DBST index can be a useful index associated with the objective measurement of insomnia. Notably, we did not conduct nocturnal polysomnography in this study. Therefore, we could not determine the relationship between the DBST index and objective sleep data. Further studies are needed to explore this relationship.

There are several limitations to this study. First, data were collected from the medical records of patients with cancer who visited the sleep clinic at a tertiary hospital that was specialized for patients with cancer. Therefore, most of the participants had psychiatric disorders or sleep disorders. This might have led to a selection bias that could limit the generalizability of the results. Furthermore, 74.7% of patients were diagnosed with insomnia disorders, and this might have influenced the results of this study. Our previous two studies explored the association between the DBST index and insomnia severity among two separate general population groups. However, in this study, the association was explored among a clinical sample of cancer patients who visited the sleep clinic for sleep disturbance issues, and this might have led to a further bias. Further study is needed to explore the associations among cancer patients in whom the proportion of insomnia disorders was not high. Second, the concept of the DBST index is not well understood, and considering that the responses were in the form of participants’ subjective answers to the physician’s questions, the index might be biased. Third, the participants’ sleep-wake cycles were assessed only on the basis of their responses in clinical interviews, without the use of objective measurement tools such as polysomnography or actigraphy. Fourth, the issue of multiple comparisons should be addressed. In this study, the observed correlations explained less than 30% of the variance due to relatively weak correlations. Considering that the concept of the DBST index is exploratory, we decided to accept the regular *P*-values rather than the adjusted *P*-values. An in-depth analysis will need to be conducted in the future. Furthermore, other sleep disorders such as obstructive sleep apnea or restless legs syndrome were not perfectly excluded, as we did not perform nocturnal polysomnography. Further studies are needed to explore the associations between the DBST index and parameters of polysomnography.

## Conclusion

We observed that the DBST index was associated with the severity of insomnia in patients with cancer. In addition, long SOL durations could be predicted by the DBST index. Notably, the DBST index could be a potential new sleep index that can predict sleep problems experienced by patients with cancer. In clinical settings, insomnia disorders or sleep problems are frequently neglected. However, sleep disturbances may influence patients’ quality of life and require further study. The DBST index is a simple way to explore patients’ insomnia severity by asking them about their desired TIB and TST. The DBST index can be useful when applied as an auxiliary index to assess the insomnia severity and sleep latency durations when assessing the sleep problems experienced by patients with cancer by.

## Data availability statement

The raw data supporting the conclusions of this article will be made available by the authors, without undue reservation.

## Ethics statement

This study protocol was approved by the Asan Institutional Review Board with the consent waiver (Approval No.: S2022-0596-0001). Written informed consent for participation was not required for this study in accordance with the national legislation and the institutional requirements.

## Author contributions

SC, HC, and JL: concept. SC, JS, EC, DL, HK, IKC, and JL: data curation. SC: formal analysis. EC, JS, SC, IKC, DL, HK, and JL: writing—original manuscript. EC, JS, JL, IKC, DL, HC, HK, and SC: review and editing of the manuscript. All authors contributed to the article and approved the submitted version.

## Supplementary material

Supplementary Figure 1 Correlation between the DBST index and **(A)** insomnia severity index and **(B)** sleep onset latency.

Supplementary Table 1 Demographic and clinical characteristics of the study subjects (*n* = 146).

Supplementary Table 2 Sleep indices of the study subjects (*n* = 146).

## References

[1] Ohayon MM. Epidemiology of insomnia: what we know and what we still need to learn. *Sleep Med Rev.* (2002) 6:97–111. 10.1053/smrv.2002.0186 12531146

[2] Winkelman JW. Insomnia disorder. *N Engl J Med.* (2015) 373:1437–44. 26444730 10.1056/NEJMcp1412740

[3] Wilson S Nutt D Alford C Argyropoulos S Baldwin D Bateson A British Association for Psychopharmacology consensus statement on evidence-based treatment of insomnia, parasomnias and circadian rhythm disorders. *J Psychopharmacol.* (2010) 24:1577–601. 10.1177/0269881110379307 20813762

[4] Qaseem A Kansagara D Forciea MA Cooke M Denberg TD Clinical Guidelines Committee of the American College of Physicians. Management of chronic insomnia disorder in adults: a clinical practice guideline from the American College of Physicians. *Ann Internal Med.* (2016) 165:125–33. 27136449 10.7326/M15-2175

[5] van der Zweerde T Bisdounis L Kyle SD Lancee J van Straten A. Cognitive behavioral therapy for insomnia: a meta-analysis of long-term effects in controlled studies. *Sleep Med Rev.* (2019) 48:101208. 31491656 10.1016/j.smrv.2019.08.002

[6] Thakral M Von Korff M McCurry SM Morin CM Vitiello MV. Changes in dysfunctional beliefs about sleep after cognitive behavioral therapy for insomnia: a systematic literature review and meta-analysis. *Sleep Med Rev.* (2020) 49:101230. 10.1016/j.smrv.2019.101230 31816582 PMC7012685

[7] Schneider MN Kovas Y Gregory AM. Dysfunctional beliefs about sleep and insomnia symptoms in early adulthood: a twin and sibling study. *J Sleep Res.* (2019) 28:e12834. 10.1111/jsr.12834 30873709

[8] Morin CM Bélanger L LeBlanc M Ivers H Savard J Espie CA The natural history of insomnia: a population-based 3-year longitudinal study. *Arch Internal Med.* (2009) 169:447–53. 10.1001/archinternmed.2008.610 19273774

[9] Hoang HTX Molassiotis A Chan CW Nguyen TH Liep Nguyen V. New-onset insomnia among cancer patients undergoing chemotherapy: prevalence, risk factors, and its correlation with other symptoms. *Sleep Breath.* (2020) 24:241–51. 10.1007/s11325-019-01839-x 31016572

[10] Chung S Youn S Choi B. Assessment of cancer-related dysfunctional beliefs about sleep for evaluating sleep disturbance in cancer patients. *Sleep Med Res.* (2017) 8:98–101. 10.17241/smr.2017.00073

[11] Youn S Kim C Lee J Yeo S Suh S Chung S. Development of dysfunctional beliefs and attitude about sleep scale for cancer patients. *Behav Sleep Med.* (2020) 18:287–97. 10.1080/15402002.2019.1578773 30789064

[12] O’Donnell JF. Insomnia in cancer patients. *Clin Cornerstone.* (2004) 6:S6–14. 10.1016/s1098-3597(05)80002-x 15675652

[13] Reynolds-Cowie P Fleming L. Living with persistent insomnia after cancer: a qualitative analysis of impact and management. *Br J Health Psychol.* (2021) 26:33–49. 10.1111/bjhp.12446 32558129

[14] Collins KP Geller DA Antoni M Donnell DM Tsung A Marsh JW Sleep duration is associated with survival in advanced cancer patients. *Sleep Med.* (2017) 32:208–12. 10.1016/j.sleep.2016.06.041 28366336 PMC5428985

[15] Lee J Cho IK Kim K Kim C Park CHK Yi K Discrepancy between desired time in bed and desired total sleep time, insomnia, depression, and dysfunctional beliefs about sleep among the general population. *Psychiatry Investig.* (2022) 19:281–8. 10.30773/pi.2021.0373 35500901 PMC9058269

[16] Chung S. The DBST index, the discrepancy between desired time in bed and desired total sleep time: the possible new sleep index predicting severity of insomnia. *Sleep Med Res.* (2022) 13:85–93. 10.17241/smr.2022.01368 PMC9874099 36713894

[17] Stores G. *Insomnia and Other Adult Sleep Problems.* Oxford: Oxford University Press (2009).

[18] Borbély AA Daan S Wirz-Justice A Deboer T. The two-process model of sleep regulation: a reappraisal. *J Sleep Res.* (2016) 25:131–43. 10.1111/jsr.12371 26762182

[19] Castelnovo A Ferri R Punjabi NM Castronovo V Garbazza C Zucconi M The paradox of paradoxical insomnia: a theoretical review towards a unifying evidence-based definition. *Sleep Med Rev.* (2019) 44:70–82. 10.1016/j.smrv.2018.12.007 30731262

[20] Şenel GB Aydın Ö Aydın ET Bayar MR Karadeniz D. Changes in sleep structure and sleep spindles are associated with the neuropsychiatric profile in paradoxical insomnia. *Int J Psychophysiol.* (2021) 168:27–32. 10.1016/j.ijpsycho.2021.07.626 34331959

[21] Rezaie L Fobian AD McCall WV Khazaie H. Paradoxical insomnia and subjective–objective sleep discrepancy: a review. *Sleep Med Rev.* (2018) 40:196–202. 29402512 10.1016/j.smrv.2018.01.002

[22] Chung S Youn S Kim C. Are you asking what time did your patients go to bed?: getting the short sleep onset latency. *Sleep Med Res.* (2018) 9:58–62.

[23] Morin CM Belleville G Belanger L Ivers H. The Insomnia Severity Index: psychometric indicators to detect insomnia cases and evaluate treatment response. *Sleep.* (2011) 34:601–8. 10.1093/sleep/34.5.601 21532953 PMC3079939

[24] Cho YW Song ML Morin CM. Validation of a Korean version of the insomnia severity index. *J Clin Neurol.* (2014) 10:210–5. 25045373 10.3988/jcn.2014.10.3.210 PMC4101097

[25] Kroenke K Spitzer RL Williams JB. The PHQ-9: validity of a brief depression severity measure. *J Gen Intern Med.* (2001) 16:606–13. 10.1046/j.1525-1497.2001.016009606.x 11556941 PMC1495268

[26] Kim K Kim H Lee J Cho IK Ahn MH Son KY Functional impairments in the mental health, depression and anxiety related to the viral epidemic, and disruption in healthcare service utilization among cancer patients in the COVID-19 pandemic era. *Cancer Res Treat.* (2021) 54:671–9. 10.4143/crt.2021.585 34583461 PMC9296928

[27] Metzger RL. A reliability and validity study of the State-Trait Anxiety Inventory. *J Clin Psychol.* (1976) 32:276–8.

[28] Han DW Lee CH Chon KK. Korean adaptation of Spielberger’s STAI (K-STAI). *Korean J Health Psychol.* (1996) 1:1–14.

[29] Herschbach P Berg P Dankert A Duran G Engst-Hastreiter U Waadt S Fear of progression in chronic diseases: psychometric properties of the Fear of Progression Questionnaire. *J Psychosom Res.* (2005) 58:505–11. 10.1016/j.jpsychores.2005.02.007 16125517

[30] Shim EJ Shin YW Oh DY Hahm BJ. Increased fear of progression in cancer patients with recurrence. *Gen Hosp Psychiatry.* (2010) 32:169–75. 10.1016/j.genhosppsych.2009.11.017 20302991

[31] Mehnert A Herschbach P Berg P Henrich G. Koch U. [Fear of progression in breast cancer patients–validation of the short form of the Fear of Progression Questionnaire (FoP-Q-SF)]. *Z Psychosom Med Psychother.* (2006) 52:274–88. 10.13109/zptm.2006.52.3.274 17156600

[32] Bijur PE Latimer CT Gallagher EJ. Validation of a verbally administered numerical rating scale of acute pain for use in the emergency department. *Acad Emerg Med.* (2003) 10:390–2. 10.1111/j.1553-2712.2003.tb01355.x 12670856

[33] Mitchell SA. Cancer-related fatigue: state of the science. *PM R.* (2010) 2:364–83. 10.1016/j.pmrj.2010.03.024 20656618

[34] Zhou ES Suh S Youn SC. Adapting cognitive-behavior therapy for insomnia in cancer patients. *Sleep Med Res.* (2017) 8:51–61.

[35] Savard J Morin CM. Insomnia in the context of cancer: a review of a neglected problem. *J Clin Oncol.* (2001) 19:895–908. 10.1200/JCO.2001.19.3.895 11157043

[36] Morin CM Vallières A Ivers H. Dysfunctional beliefs and attitudes about sleep (DBAS): validation of a brief version (DBAS-16). *Sleep.* (2007) 30:1547–54. 10.1093/sleep/30.11.1547 18041487 PMC2082102

[37] Dahiya S Ahluwalia MS Walia HK. Sleep disturbances in cancer patients: underrecognized and undertreated. *Cleve Clin J Med.* (2013) 80:722–32. 10.3949/ccjm.80a.12170 24186891

[38] Morin CM. Cognitive-behavioral approaches to the treatment of insomnia. *J Clin Psychiatry.* (2004) 65(Suppl 16):33–40. 15575803

[39] Espie CA. Insomnia: conceptual issues in the development, persistence, and treatment of sleep disorder in adults. *Annu Rev Psychol.* (2002) 53:215–43. 10.1146/annurev.psych.53.100901.135243 11752485

[40] Borbely AA Daan S Wirz-Justice A Deboer T. The two-process model of sleep regulation: a reappraisal. *J Sleep Res.* (2016) 25:131–43. 10.1111/jsr.12371 26762182

