# Supplementary figures and images for: Discrepancy between desired time in bed and desired total sleep time in patients with cancer: The DBST index and its relationship with insomnia severity and sleep onset latency
Source: Front Psychiatry. 2023 Jan 11;13:978001. doi: 10.3389/fpsyt.2022.978001 (PMC9874099; doi:10.3389/fpsyt.2022.978001)

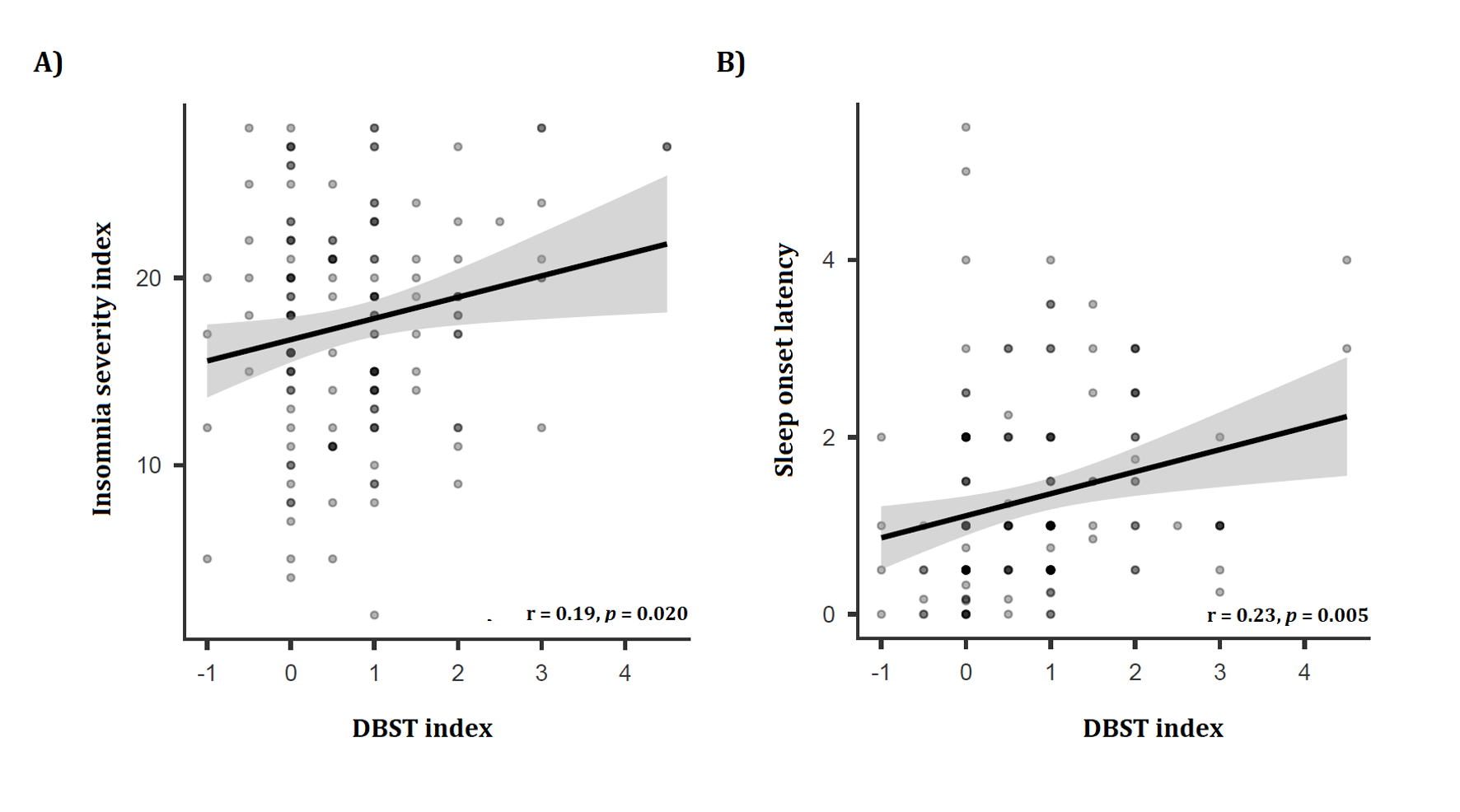

Supplement: Supplementary Figure 1 — Correlation between the DBST index and (A) insomnia severity index and (B) sleep onset latency. [file Image_1.tif]
