# Supplementary material for: Discrepancy between desired time in bed and desired total sleep time in patients with cancer: The DBST index and its relationship with insomnia severity and sleep onset latency
Source: Front Psychiatry. 2023 Jan 11;13:978001. doi: 10.3389/fpsyt.2022.978001 (PMC9874099; doi:10.3389/fpsyt.2022.978001)
Supplement: Supplementary Table 1 — Demographic and clinical characteristics of the study subjects (n = 146). [file Table_1.docx]

**Supplementary Table 1.** Demographic and clinical characteristics of the study subjects (n=146)

| Variable | N (%), Mean ± SD |
| --- | --- |
|  |  |
| **Gender, female** | 108 (74.0%) |
| **Age (years)** | 56.7 ± 11.6 |
| **Marital status** |  |
| Unmarried | 11 (7.5%) |
| Married | 132 (90.4%) |
| Divorced | 3 (2.1%) |
| **Psychiatric illness, presence** | 144 (98.6%) |
| Insomnia disorders | 109 (74.7%) |
| Depressive disorders | 17 (11.6%) |
| Anxiety disorders | 8 (5.5%) |
| Adjustment disorder | 3 (2.1%) |
| Somatic symptom and related disorders | 2 (1.4%) |
| Specific phobia | 1 (0.7%) |
| Others | 4 (2.7%) |
| **Cancer types** |  |
| Solid tumor | 137 (93.8%) |
| Breast cancer | 65 (44.5%) |
| Gastrointestinal, hepatobiliary, and pancreatic cancer | 37 (25.3%) |
| Other malignancy | 35 (24.0%) |
| Hematologic malignancy | 9 (6.2%) |
| **Cancer stages (among TNM classification available patients, N = 128)** |  |
| Stage I, II, III | 106 (82.8%) |
| Stage IV | 22 (17.2%) |
| **Surgery within 3 months** | 42 (28.8%) |
| **Current cancer treatment, presence** |  |
| Chemotherapy | 46 (31.5%) |
| Radiation therapy | 18 (12.3%) |
| Hormone therapy | 41 (28.1%) |
| Immune/target therapy | 7 (4.8%) |
| **Questionnaires, score** |  |
| Insomnia Severity Index (ISI) | 17.6 ± 5.9 |
| Patients Health Questionnaire-9 (PHQ-9) | 11.1 ± 6.3 |
| State subcategory of the State and Trait Anxiety Inventory (STAI-S) | 41.8 ± 8.9 |
| Fear of Progression questionnaire-short form (FoP-Q-SF) | 35.3 ± 12.3 |
| Cancer-related Dysfunctional Beliefs about Sleep (C-DBS) | 13.6 ± 4.9 |
| Pain, numeric rating scale | 3.8 ± 2.9 |

**Supplementary Table 2.** Sleep indices of the study subjects (n=146)

| **Variable** | **Mean ± SD** |
| --- | --- |
|  |  |
| **Time variables** |  |
| Bedtime | 10:42 ± 1:12 PM |
| Sleep onset time | 11:59 ± 1:18 PM |
| Wake-up time | 6:36 ± 1:24 AM |
| **Duration variables** |  |
| Sleep onset latency, SOL (min) | 90.0 ± 60.0 |
| Time in bed, TIB (h) | 7.9 ± 1.5 |
| Duration from wake-up time to bedtime, WTB (h) | 16.1 ± 1.5 |
| **DBST** |  |
| Desired hours of time in bed (desired TIB) | 7.4 ± 1.0 |
| Desired hours of total sleep time (desired TST) | 6.6 ± 1.2 |
| DBST index | 0.8 ± 1.0 (-1.0 ~ 4.5) |
